# Effects of Different Interventions Using Taekwondo, Boxing, and Elastic Band Training on Body Composition and Physical Function in Chilean Older Women: A Randomized Controlled Trial

**DOI:** 10.3390/life15071049

**Published:** 2025-06-30

**Authors:** Edgar Vásquez-Carrasco, Jordan Hernandez-Martinez, Izham Cid-Calfucura, Eduardo Guzmán-Muñoz, Camila Ruiz, Camila Baeza, María José Márquez, Tomás Herrera-Valenzuela, Braulio Henrique Magnani Branco, Eduardo Carmine-Peña, Paulina Sepúlveda, Cristian Sandoval, Pablo Valdés-Badilla

**Affiliations:** 1 School of Occupational Therapy, Faculty of Psychology, University of Talca, Talca 3465548, Chile; edgar.vasquez@utalca.cl; 2 Centro de Investigación en Ciencias Cognitivas, Faculty of Psychology, Universidad de Talca, Talca 3465548, Chile; 3 VITALIS Longevity Center, Universidad de Talca, Talca 3465548, Chile; 4 Department of Physical Activity Sciences, Universidad de Los Lagos, Osorno 5290000, Chile; jordan.hernandez@ulagos.cl; 5 Department of Education, Faculty of Humanities, Universidad de La Serena, La Serena 1700000, Chile; 6 Escuela de Ciencias del Deporte y Actividad Física, Facultad de Salud, Universidad Santo Tomás, Santiago 8320000, Chile; izham.cid@gmail.com; 7 School of Kinesiology, Faculty of Health, Universidad Santo Tomás, Talca 3530000, Chile; eguzmanm@santotomas.cl; 8 School of Kinesiology, Faculty of Health Sciences, Universidad Autónoma de Chile, Talca 3530000, Chile; 9 Physical Education Pedagogy, Universidad de Los Lagos, Osorno 5290000, Chile; camilavaleria.ruiz@alumnos.ulagos.cl (C.R.); camilafernanda.baeza@alumnos.ulagos.cl (C.B.); mariajose.marquez2@alumnos.ulagos.cl (M.J.M.); 10 Department of Physical Activity, Sports and Health Sciences, Faculty of Medical Sciences, Universidad de Santiago de Chile (USACH), Santiago 8370003, Chile; tomas.herrera@usach.cl; 11 Postgraduate Program in Health Promotion, Cesumar University, Maringá 87050-390, Paraná, Brazil; braulio.branco@unicesumar.edu.br; 12 Carrera de Medicina, Facultad de Medicina, Universidad de La Frontera, Temuco 4811230, Chile; e.carmine01@ufromail.cl; 13 Departamento de Ciencias Preclínicas, Facultad de Medicina, Universidad de La Frontera, Temuco 4811230, Chile; paulina.sepulveda@ufrontera.cl; 14 Escuela de Tecnología Médica, Facultad de Salud, Universidad Santo Tomás, Los Carreras 753, Osorno 5310431, Chile; 15 Departamento de Medicina Interna, Facultad de Medicina, Universidad de La Frontera, Temuco 4811230, Chile; 16 Department of Physical Activity Sciences, Faculty of Education Sciences, Universidad Católica del Maule, Talca 3530000, Chile; 17 Sports Coach Career, Faculty of Life Sciences, Universidad Viña del Mar, Viña del Mar 2520000, Chile

**Keywords:** aging, martial arts, muscle strength, postural balance, sports medicine

## Abstract

**Background**: Interventions involving Olympic combat sports, such as Taekwondo (TKD) and Boxing (BOX), represent innovative approaches for promoting health in older people. Elastic bands training (EBT), by contrast, is a safe and cost-effective method that has demonstrated positive effects on functional physical parameters in this population. This study aimed to compare the effects of TKD and BOX interventions, relative to EBT, on body composition and physical function in Chilean older women. **Methods**: This randomized controlled trial comprised three parallel groups: TKD (*n* = 10), BOX (*n* = 10), and EBT (*n* = 10). Participants in each group underwent pre- and post-intervention assessments following two 60 min sessions per week over an eight-week period. **Results**: Multiple comparisons revealed significant advantages for EBT over TKD and BOX in maximal isometric handgrip strength for both the dominant (*p* < 0.001; d = 0.967, large effect) and non-dominant (*p* < 0.001; d = 0.641, moderate effect) hands. Conversely, significant improvements in Timed Up-and-Go performance were observed in the TKD and BOX groups compared to EBT (*p* < 0.001; d = 2.071, large effect). All groups showed significant within-group improvements in the 30 s chair stand test (*p* < 0.001; d = 0.095, large effect). No significant changes were found in body fat percentage, fat-free mass, back scratch test, sit-and-reach test, or 2 min step test across groups. **Conclusions**: Although no substantial differences were observed between groups for most variables, TKD and BOX interventions significantly reduced Timed Up-and-Go times, whereas EBT enhanced maximal isometric handgrip strength in both hands. These findings highlight the distinct functional benefits of each intervention modality for older Chilean women.

## 1. Introduction

During aging, natural biological changes in body composition occur, including an increase in body fat percentage (BFP) and a decrease in fat-free mass (FFM) [1,2,3]. These changes are accompanied by declines in physical function, such as reductions in muscle strength, balance, flexibility, and cardiorespiratory fitness [1,4,5,6]. Collectively, these factors contribute to an elevated risk of falls and functional dependence [5,7,8], a risk that is higher in women than in men, both globally and in Chile [7]. Physical inactivity is a major determinant that exacerbates alterations in body composition and physical function among Chilean older women [8]. Conversely, physical activity interventions have been shown to improve these age-related impairments in older women [9,10,11,12].

Traditional multimodal physical activity programs—including walking, resistance training, and aquatic exercise—have demonstrated positive effects on physical function and body composition in older women. These interventions contribute to better performance in activities of daily living, enhance self-esteem, and improve quality of life in this population [11,12,13,14]. However, their implementation often depends on favorable climate conditions and entails considerable economic costs [15]. As alternative strategies, elastic band training (EBT) and Olympic combat sports (OCS) offer promising options [13,14]. A recent meta-analysis reported significant benefits of EBT compared to both active and inactive control groups, with improvements noted in the 30 s chair stand test, sit-and-reach flexibility, and Timed Up-and-Go (TUG) performance [13]. However, no significant changes were observed in BFP or FFM, as the results did not reach statistical significance. Similarly, OCS interventions, such as taekwondo (TKD) and boxing (BOX), are among the most widely applied strategies for older adults [14,15]. According to a systematic review [16], OCS programs significantly reduce fall risk and improve balance when compared to both active and inactive control groups.

Although evidence exists regarding the effects of both Olympic combat sports (OCS) and elastic band training (EBT) on indicators of body composition and physical function [17,18], it is essential to conduct comparisons through randomized controlled trials (RCTs) with active comparator groups to more accurately determine the specific effects of each intervention [19]. For example, a three-arm RCT conducted in healthy older women reported significant improvements in Maximal Isometric Handgrip Strength (MIHS) and 30 s chair stand performance in favor of EBT compared to multicomponent training and group-based dance interventions [20]. Similarly, a two-arm RCT involving older women with sarcopenia found that EBT significantly improved fat-free mass (FFM), MIHS in both dominant and non-dominant hands, and Timed Up-and-Go (TUG) performance relative to group-based dance [21]. A multi-arm RCT in older women also demonstrated that taekwondo (TKD) led to significant improvements in 30 s chair stand, sit-and-reach, TUG, and MIHS in the non-dominant hand compared to multicomponent training, walking exercise, and an inactive control group (*p* < 0.001) [22]. In older adults with Parkinson’s disease, a two-arm RCT by Combs et al. [23] found that boxing (BOX) interventions significantly improved 6 min walk test performance compared to multicomponent training.

While prior studies have explored the individual effects of TKD, BOX, and EBT on body composition and physical function [20,24], no study has directly compared these three interventions in the same trial. Given the importance of RCTs with active comparators and the increased statistical power provided by trials with three or more arms [24,25], the aim of this study was to evaluate the effects of TKD and BOX interventions, relative to EBT, on body composition and physical function in Chilean older women. It was hypothesized that all three interventions would improve lower-body muscle strength, with EBT alone enhancing upper-body muscle strength [20,24,26]. Conversely, only TKD and BOX were expected to improve dynamic balance in physically inactive older women.

## 2. Materials and Methods

### 2.1. Study Design

The TKD, BOX, and EBT groups constituted the three parallel arms of this RCT, with 10 participants assigned to each group (*n* = 10 each). The study employed a repeated measures design with a quantitative approach and incorporated double-blind methods, in which both participants and evaluators were blinded. Randomization was conducted using the Research Randomizer website (https://www.randomizer.org; accessed on 17 January 2025). Concealed allocation was ensured through the use of sealed envelopes. The trial adhered to CONSORT guidelines [27], and the study protocol was registered at ClinicalTrials.gov (identifier: NCT06780020; https://clinicaltrials.gov/search?cond=NCT06780020; registered 17 January 2025).

The intervention period spanned 8 weeks, comprising 16 sessions in total, with two 60 min sessions held weekly on Tuesdays and Thursdays. Outcome assessments included the 30 s chair stand test, Timed Up-and-Go (TUG) test, sit-and-reach test, back scratch test, 2 min step test, and measurements of body fat percentage (BFP), fat-free mass (FFM), and maximal isometric handgrip strength (MIHS) of both the dominant and non-dominant hands. All evaluations were performed at the same location (sports facility and social headquarters) between 14:00 and 16:00, with the same evaluators conducting pre- and post-intervention measurements while controlling for environmental factors such as temperature. No participants reported pain prior to the evaluations or during the training sessions, and no musculoskeletal or cardiorespiratory adverse events occurred during the intervention.

### 2.2. Participants

**Figure 1:**
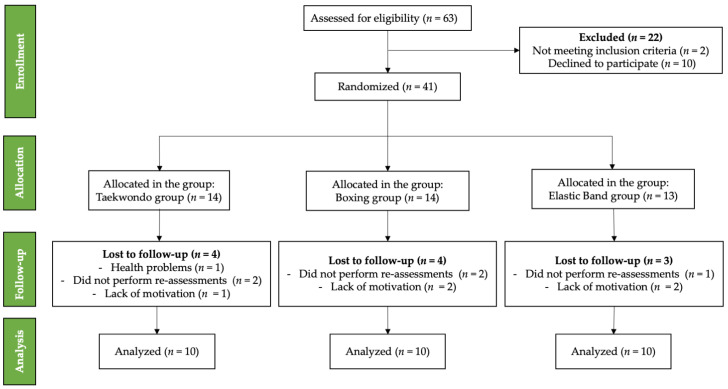
Flowchart for older women’s enrollment, allocation, follow-up, and analysis.

Initially, 63 older women took part in the intervention. The sample size calculation indicated that 10 individuals per group was the ideal quantity. The GPower software (version 3.1.9.6, Franz Faul, Universität of Kiel, Kiel, Germany) was used to determine statistical power. Considering an alpha level of 0.05, a power of 95%, and a predicted loss of 10%, a prior study [17] found that a mean difference of 0.50 s with a standard deviation of 0.93 s in the TUG was the minimum difference required for significant clinical relevance. Similarly, an average difference of 3.46 replicates in 30 s chair stand test was used as the minimum difference required for substantial clinical relevance, with a standard deviation of 3.38 replicates. Inclusion criteria: (i) women aged 65 to 75 years; (ii) able to comprehend and follow instructions contextualized through basic directives; (iii) independent, as measured by a score of at least 43 points on the Chilean Ministry of Health’s Preventive Medicine Examination for Older People [28]; (iv) who lead a sedentary and/or physically inactive lifestyle; and (v) meeting the attendance requirement of at least 85% at the intervention. The following factors were considered when determining the exclusion criteria: (i) having a disability; (ii) undergoing physical rehabilitation or suffering from musculoskeletal injuries that hinder their ability to perform their daily activities; and (iii) being either permanently or temporarily unable to engage in physical activity. Participants who met the inclusion criteria also had to complete at least 85% of the training sessions and attend all assessment sessions to be included in the final analysis. The inclusion criteria are summarized in Figure 1.

All participants granted consent for data usage and processing by signing an informed consent form, which authorized the use of personal data for scientific purposes. The scientific ethics committee of Universidad Católica del Maule in Chile approved the protocol, formulated in accordance with the Declaration of Helsinki (Approval Number: N°29-2022).

### 2.3. Measurements and Interventions

#### 2.3.1. Physical Performance

Previous studies have recommended the use of MIHS testing [29]. The most suitable testing position was determined to be seated, with the forearm and wrist maintained in a neutral position, the elbow flexed at 90° and kept close to the torso, the spine properly aligned, and the shoulder relaxed in a neutral position. A handheld dynamometer (Jamar^®^, PLUS+, Sammons Preston, Patterson Medical, Warrenville, IL, USA) was used to perform the measurements. To accommodate varying hand sizes and ensure effective engagement of the metacarpophalangeal and interphalangeal joints, the dynamometer was set to the first position, allowing contact between the thumb and the proximal phalanx of the index finger. Participants were given a 120 s rest period between each of the three repetitions per hand.

Three repetitions were evaluated to retrieve the best performance executed on the 30 s chair stand test [30]. Its purpose was to evaluate the lower limbs’ muscular strength, and it is executed while seated in a chair, with the arms resting across the chest, for 30 s.

The TUG test was conducted in accordance with earlier guidelines [30]. An arm-supported chair requires the user to exit, go three meters down a hallway, turn around, and then return to the chair. Following completion of the trials, the best time out of three attempts was recorded. Timing was measured by two assessors using single-beam photocells (Brower Timing System, Draper, UT, USA), and the top three efforts were included in the statistical analysis.

Lower limb flexibility was assessed through the sit-and-reach test, following the protocol described previously [31]. Participants sat in a chair with a fixed backrest, extending one leg forward while keeping the other bent and resting on the floor. The test was performed on the same leg in both the pre- and post-intervention evaluations, although participants were allowed to select either the left or right leg based on comfort. Two attempts were made, and the higher of the two measurements was recorded as the result, with adjustments made if the posture required correction.

For the back scratch test (upper limb flexibility), the participant placed one hand on the shoulder and one hand towards the middle of the back, maintaining the position. The evaluator measured the amount of cm (+ or −) between the extended ring fingers of both hands [30]. These deviations from the original method may have affected measurement accuracy and contributed to the observed day-to-day variability in the results, thus introducing potential assessment bias. Therefore, the findings from the back scratch test should be interpreted with caution and in the context of this methodological limitation.

The 2 min step test was used to measure cardiorespiratory fitness [27]. Participants were told to stand upright, and colorful tape was used to indicate the midway between the patella and pelvic bones on a wall. The number of repeats within 2 min was recorded as the participants stepped with their knees elevated over the designated spot [30].

#### 2.3.2. Anthropometric and Sociodemographic Parameters

All anthropometric measures were completed following the International Society for the Advancement of Kinanthropometry (ISAK) criteria [32]. The ISAK criteria were followed for each measurement [33].

#### 2.3.3. Intervention

**Figure 2:**
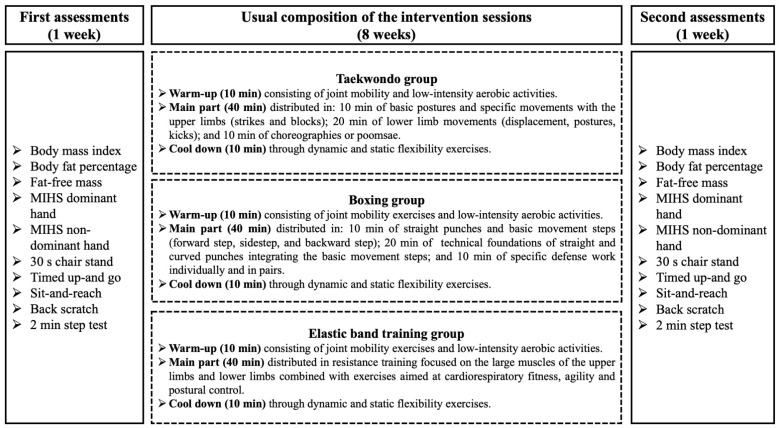
Measurements and regular sessions of the intervention. MIHS: maximal isometric handgrip strength.

Following the guidelines of earlier research, the interventions (TKD, BOX, and EBT) were carried out [21,24,34]. The eight-week (16-session) regimens included a warm-up that included low-intensity aerobic and joint mobility exercises, a main 40 min session that included TKD, BOX, or EBT, and a 10 min cool-down that included static flexibility exercises. A summary of the intervention’s measurements and planned sessions is shown in Figure 2.

The core of the TKD training program involved non-contact exercises, structured into two segments: a 10 min session focused on basic stances and upper limb techniques (including blocks and strikes), followed by 20 min dedicated to lower limb techniques, such as stances, movements, and kicks. These drills were practiced both individually and with partners, utilizing TKD shields and pads in some instances. An additional 10 min was allocated to choreographed sequences, specifically the Kibom Poomsae and Il Jang patterns [26].

In the initial 2 weeks, participants completed (3 sets × 8 repetitions × 2 min of recovery between each set) with whole-body techniques [24,26]. From week 3 to week 5, the training volume was increased (4 sets × 8 repetitions × 2 min of recovery between each set). In week 6, (4 sets × 12 repetitions), the volume being similar in weeks 7 and 8, the rest duration was reduced to 90 s.

The BOX program consisted of non-contact exercises (main part) [35], which were divided into 10 min of straight punches and basic movement steps (forward step, sidestep, and backward step) and 20 min of technical foundations of straight and curved punches, integrating the basic movement steps. In addition, specific defense work was carried out individually and in pairs for 10 min. The specific elements of each class varied beyond the 4 weeks, with a progression from learning simple exercises and simple drills, and foundations, to learning more complex movements and combinations. Throughout the first 2 weeks of the program, participants performed three sets of eight repetitions focusing on fundamental movements and isolated punching techniques, with a two-minute rest interval between sets [36]. From weeks 3 to 5, the training volume was increased to four sets of eight repetitions, now incorporating punch combinations consisting of three to four consecutive strikes, while maintaining the same rest duration. In week 6, the number of sets remained at four, while the number of repetitions per set was raised to 12. Finally, the rest period was reduced to 90 s between weeks 7 and 8, maintaining the number of sets and repetitions (4 × 12).

Previous studies have demonstrated the safety and efficacy of EBT for older people [17,19], employing TheraBand^®^ resistance bands produced by the Hygienic Corporation (Akron, OH, USA). The training intensity was regulated through color-coded bands—yellow, red, green, blue, black, silver, and gold—each representing a specific level of tension. Resistance training intensity ranged from moderate to intense (5 to 8 points) on the OMNI Resistance Exercise Scale (OMNI-RES) [37]. Leg press, ankle eversion, ankle dorsiflexion, knee extension, knee flexion, and hip flexion were among the six lower limb muscle strength exercises performed, along with six upper limb movements (pull-up, pullback, shoulder abduction, biceps curl, triceps, and forearm). In order to reach a 10-repetition maximum (10 RM) for activities that targeted the upper and/or lower limbs, the older women participants started training with the lowest resistance band (yellow). After finishing a 10 RM, the participant advanced to bands of increasing resistance until no more advancement was feasible. The last effective resistance band was used to initiate the training regimen. During each training session, participants performed two sets of exercises at 100% of their 10 RM intensity, with one minute of rest between each set. During an 8-week intervention period, total body exercises were performed at a volume of 2 sets × 10 to 15 repetitions. The band provided the greatest resistance, and maximal strength was measured at 10 RM. The length of the band was halved, and the program was maintained for the full 8 weeks if a participant was unable to progress to a higher resistance and completed 10 RM. Every 4 weeks, those who successfully achieved 10 RM moved on to the next band color. Details on the dosing of the interventions, as well as intensities, are presented in detail in Table 1.

### 2.4. Statistical Analysis

GraphPad Prism version 9.0 was used to do the statistical analysis, which concentrated on descriptive and inferential data. Descriptive statistics included the calculation of means, standard deviations, 95% confidence intervals, and relative frequencies, depending on the type of variable analyzed. The data distribution’s normality was evaluated using the Shapiro–Wilk test. The interaction between group and time was then examined using a two-factor mixed ANOVA with repeated measurements. Bonferroni post hoc tests were used to assess intra-group differences (baseline vs. after) and inter-group differences (TKD vs. BOX vs. EBT) in situations where the group × time interaction was significant. Effect sizes for Eta-squared pairwise comparisons were determined using Cohen’s d [38] and classified as small (≥0.2), medium (≥0.5), or large (≥0.8). For every analysis, the α level of 0.05 was used to determine statistical significance.

## 3. Results

According to the baseline outcomes, the mean age of the older women under analysis was 71.8 ± 4.29 years. Furthermore, 15% of them held a bachelor’s degree, 40% had a secondary academic degree, and 45% had a primary academic level. Table 2 also reveals that 66% were married, 20% were separated, and 14% were widowed.

Table 3 presents the baseline and after-intervention results for body composition and physical function variables in the TKD, BOX, and EBT groups. Prior to the intervention, we assessed the homogeneity of the groups using one-way ANOVA for all baseline variables. No significant differences were found between TKD, BOX, and EBT groups in any of the physical function or body composition measures at baseline (*p* > 0.05).

The two-way mixed ANOVA analysis revealed significant time × group interactions for MIHS dominant hand (F_(2,31)_ = 55.940; *p* < 0.001; ηp^2^ = 0.783), MIHS non-dominant hand (F_(2,31)_ = 29.180; *p* < 0.001; ηp^2^ = 0.652), and TUG (F_(2,31)_ = 21.770; *p* < 0.001; ηp^2^ = 0.584). However, no significant time × group interactions were observed for FFM (F_(2,31)_ = 0.306; *p* = 0.738; ηp^2^ = 0.019), BFP (F_(2,31)_ = 2.588; *p* = 0.091; ηp^2^ = 0.143), 30 s chair stand (F_(2,31)_ = 1.636; *p* = 0.211; ηp^2^ = 0.095), back scratch (F_(2,31)_ = 2.665; *p* = 0.084; ηp^2^ = 0.139), sit-and-reach (F_(2,31)_ = 1.882; *p* = 0.168; ηp^2^ = 0.102), and 2 min step test (F_(2,31)_ = 1.982; *p* = 0.166; ηp^2^ = 0.113).

**Figure 3:**
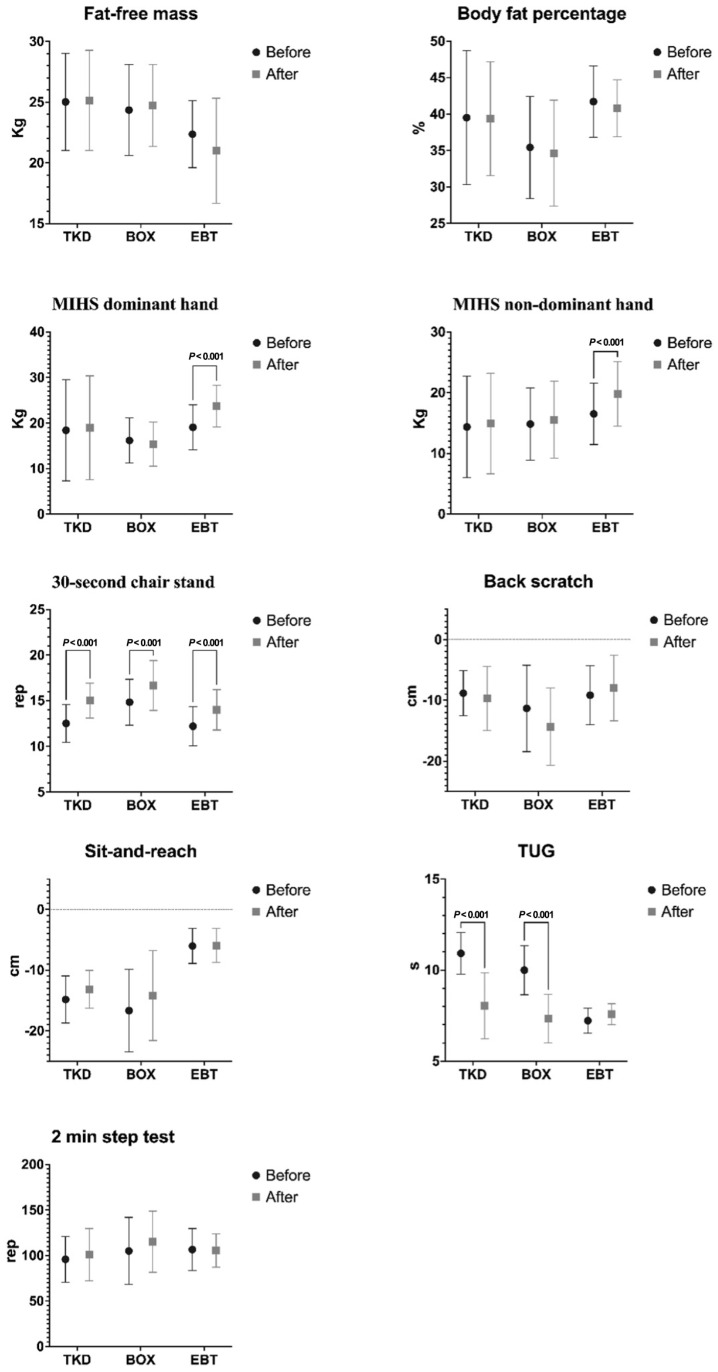
Multiple intragroup and intergroup comparisons for body composition and physical function. BOX: boxing; EBT: elastic band training; MIHS: maximal isometric handgrip strength; TKD: taekwondo; TUG: timed Up-and-Go.

Figure 3 presents the results of the multiple within-group and between-group comparisons for all assessed variables, including FFM, BFP, MIHS for both dominant and non-dominant hands, the 30 s chair stand, back scratch, sit-and-reach, TUG, and the 2 min step test. Following the intervention, the EBT group demonstrated significant improvements in MIHS for both the dominant hand (*p* < 0.001; ES = 0.967, large effect) and the non-dominant hand (*p* < 0.001; ES = 0.641, medium effect). In contrast, no significant changes were observed for these variables within the TKD or BOX groups. Significant improvements in TUG performance were noted in the TKD and BOX groups (*p* < 0.001; ES = 1.944, large effect), whereas no significant improvement was found in the EBT group. No between-group differences were detected in any of the three variables that showed significant interaction effects (MIHS dominant hand, MIHS non-dominant hand, and TUG).

## 4. Discussion

The purpose of this study was to assess the effects of TKD, BOX, and EBT on body composition and physical function in Chilean older women. While the EBT group demonstrated notable improvements in MIHS for both dominant and non-dominant hands, the TKD and BOX groups showed notable gains in the TUG test. By contrast, no discernible changes were observed in any group for the 30 s chair stand, back scratch, sit-and-reach, 2 min step, or FFM tests. These findings partially support our hypothesis: TKD and BOX improved dynamic balance as reflected in TUG performance, and EBT enhanced upper-limb muscle strength, while none of the interventions produced significant improvements in lower-limb muscle strength.

Our study did not report significant improvements in FFM or BFP through TKD, BOX, or EBT interventions. These findings are consistent with previous data [26], which observed no significant changes in BFP (*p* = 0.31) or FFM (*p* = 0.62) in apparently healthy older women following 8 weeks of TKD, or multicomponent training performed three times per week, with each session lasting 60 min at an intensity of 50–70% of participants’ maximum heart rate (HRmax). Similarly, a more recent study [24] found no significant improvements in BFP (*p* = 0.525) or FFM (*p* = 0.172) after a 16-week intervention combining TKD, multicomponent training, and walking exercise, also conducted three times per week for 60 min per session at 50–70% HRmax. Our findings align with those reported in [38], which found no significant increases in FFM (*p* = 0.60) compared to an inactive control group in older adults after an 8-week EBT intervention conducted twice weekly for 60 min. This contrasts with the findings of ref. [22], which reported significant increases (*p* < 0.001) in FFM following 12 weeks of EBT performed three times per week in older women with sarcopenia, compared to group-based dance.

The differences in findings across these studies can be attributed, in part, to participants’ dietary habits, as not all studies incorporated dietary guidance to complement the interventions. There is a general tendency for older populations to consume ultra-processed foods, which negatively affects body composition and may contribute to the development of metabolic syndrome [39]. Current evidence suggests that physical activity combined with nutritional plans providing optimal protein intake (1.2–1.6 g/kg of body weight/day) can lead to improvements in body composition in older adults [40]. Therefore, interventions targeting body composition in this population should integrate nutritional education and strategies to promote healthier eating habits.

Secondly, the characteristics of the study samples should be considered. For example, ref. [22] reported significant FFM gains (*p* < 0.001) through EBT in older women with sarcopenia, who may have been more responsive to intervention due to their anthropometric profiles compared to samples of apparently healthy older adults. Finally, our twice-weekly intervention was likely insufficient to produce significant improvements in body composition. The heterogeneity of study designs, particularly the lack of nutritional monitoring, limits the ability to draw definitive conclusions. Future research should explore TKD, BOX, and EBT interventions with increased training frequency and controlled dietary intake to validate these findings.

Significant improvements in MIHS were observed in both the dominant and non-dominant hands within the EBT group, whereas no notable changes were identified in participants from the TKD and BOX groups. These findings are consistent with what was reported by previous data [22], where documented significant gains in non-dominant hand MIHS (*p* < 0.001) following an 8-week EBT intervention (2 sessions per week, 60 min each) in a sample of apparently healthy older women. Comparable results were reported in [41], which found significant improvements (*p* < 0.05) in MIHS in both hands after a 12-week EBT program conducted three times per week for 60 min per session, in comparison to an inactive control group. Similarly, significant enhancements (*p* < 0.01) in MIHS for both dominant and non-dominant hands following a 12-week EBT protocol (3 sessions per week, 60 min each) were reported, compared to a group-based dance program, in older women diagnosed with sarcopenia [22]. Different from that reported in ref. [26], where significant improvements for the non-dominant hand (*p* = 0.013) in TKD and multicomponent training (*p* = 0.006) were observed after 8 weeks of training with a frequency of 3 sessions per week of 60 min duration. Similar to the findings of ref. [42], significant increases in MIHS in the dominant hand (*p* = 0.03) were observed through TKD for 12 weeks at a frequency of 3 sessions per week lasting 60 min in older women with stage 2 hypertension compared to an inactive control group.

In this context, there is evidence of improvements in MIHS for the dominant and non-dominant hands in older people through EBT and TKD interventions. The improvements through EBT in our study could be explained by the variable resistance stimulus generated during the range of motion of a particular exercise [43]. For example, at the beginning of the movement, the resistance imposed by the elastic band is low; this allows for high-speed execution, while at the end of the movement, the resistance increases, challenging the subjects to maintain a full range of motion [43]. This variable resistance could favor brain adaptations that enhance the various expressions of muscle strength [43]. Furthermore, it has been mentioned that variable resistance training through elastic bands can improve motor unit conduction speed and the muscles’ activation during the exercise compared to dumbbells [44]. On the other hand, it is striking that we did not identify improvements in MIHS for the dominant and non-dominant hands through TKD, given that it has been reported that it can induce improvements through the constant execution of specific techniques with the upper limb [26,41]; specifically, it has been reported that prolonged time actions exerting isometric force by clenching the fists could generate an increase in the strength of the forearm muscles [26], a fact that could be similar to what occurred in BOX practice. In this context, not finding improvements in MIHS for TKD group in our study could also be attributed to the shorter duration of our training program (8 weeks) compared to other studies [24,26,42], and that they had a higher frequency (3 times per week) compared to our study of only 2 times per week. Therefore, the improvements in MIHS through TKD could follow a dose–response relationship, with positive adaptations and higher training volumes. This could also be extrapolated to BOX programs; however, further research with BOX in apparently healthy older people is needed to confirm our findings, since comparisons with populations with different pathologies can lead to bias in the analyses.

No significant changes were observed in the 30 s chair stand test across the TKD, BOX, and EBT groups. These findings contrast with those reported in [22], which showed significant improvements (*p* = 0.001) in this test among healthy older women following an 8-week TKD intervention consisting of 24 sessions lasting 60 min each. Similarly, ref. [41] reported notable gains (*p* < 0.01) in older women with arterial hypertension after 12 weeks of TKD training, conducted three times per week with 90 min sessions. Furthermore, ref. [24] also documented significant increases (*p* = 0.017) in 30 s chair stand performance after a 16-week TKD program, involving three weekly sessions of 60 min each, in a sample of apparently healthy older women. Unlike that reported in ref. [45], which did not identify changes in favor of TKD in older women with depression (*p* = 0.347) after a 12-week training program with a frequency of 3 times per week and a duration of 60 min per session. On the other hand, our results are different from those reported [17] in a meta-analysis, which reported significant improvements in the 30 s chair stand (*p* = 0.04) in favor of EBT in older people. In this context, our study had a lower training volume than the aforementioned studies since it had a weekly frequency of 2 times per week and a duration of 8 weeks. Given the existing evidence with improvements in the 30 s chair stand test through interventions with TKD and EBT in older people, we can attribute our findings to the lower training volume in the TKD, BOX, and EBT programs, in addition to not controlling the intensity of TKD and BOX training, where TKD interventions have generally used a moderate to vigorous intensity with HRmax values between 50% and 70% in older people [24]. Regarding BOX interventions, the body of evidence is still under development, so studies are needed to identify the effects of BOX on the physical function of older women.

The TKD and BOX groups showed considerable gains in the TUG test, whereas the EBT group showed no discernible improvements. This is comparable to the findings of [24], which found that TKD significantly improved TUG (*p* < 0.001) in seemingly healthy older women after 16 weeks of training at a frequency of three sessions per week lasting 60 min. Similar findings were reported in [45], which found that a 12-week TKD intervention significantly improved TUG (*p* < 0.05) in older people with depression and dementia when compared to an inactive control group. Additionally, compared to a regular gym group, older people who used EBT saw a substantial decrease in TUG time (*p* = 0.04), according to [46]. Comparable to the findings in [22], which found that EBT significantly (*p* < 0.001) improved TUG performance in older women with sarcopenia when compared to group-based dance. The TUG test assesses dynamic balance and functional mobility over 3 m distances [47]. A shorter execution time (<10 s) is strongly associated with gait independence in older people [47]. According to our results, the distinct kicking motions in TKD and the continuous punch displacements in BOX challenged older people’s centers of pressure [24]. This dynamic interaction could have stimulated proprioceptive feedback mechanisms, improving postural control during the movements [48]. This is corroborated by the fact that the force for both actions originates from the force exerted by the lower limbs against the ground, which is then transferred from the legs to the target [43]. It is possible that these continuously created forces successfully raised the torque during gait and the function of the extensor muscles of the lower limbs [44]. After both interventions, the plantar flexor muscles, which play a crucial role in the propulsive impulse at the end of the stance phase of gait, might have been able to exert more force with better postural control [44].

No significant improvements were found in lower limb flexibility in the sit-and-reach test for the TKD, BOX, and EBT groups. This is different from that reported in [24], which reported significant improvements in the sit-and-reach test (*p* < 0.001) through TKD after 16 weeks of training with a frequency of 3 sessions per week, with a duration of 60 min per session in apparently healthy older women. Similar to that reported [17] in a meta-analysis in older people with EBT interventions, significant improvements were observed in the sit-and-reach test (*p* = 0.04). Regarding our findings, we expected an improvement in range of motion through TKD and EBT, given that the exercises were performed in a greater range of motion. However, longer duration TKD and EBT interventions (minimum 16 weeks) may be necessary to induce a reduction in passive tension and stiffness of the tissues surrounding the lower limb joints and improve lower limb flexibility in apparently healthy older women [45]. Further research is needed to analyze the minimum training intensity, volume, and frequency of the BOX intervention to improve lower limb flexibility in older people.

The TKD, BOX, and EBT groups did not significantly increase their upper limb flexibility on the back scratch test. Other meta-analyses of EBT therapies in older people revealed no significant changes in favor of EBT in the back scratch test (*p* = 0.42) [17]. In a similar vein, ref. [26] found that older women who received TKD for 8 weeks and 24 sessions of 60 min each did not significantly improve on the back scratch test (*p* = 0.05). This is comparable to the findings of ref. [24], which were observed in seemingly healthy older women who, following 16 weeks of TKD training at a frequency of three times per week and lasting 60 min each session, did not significantly improve in the back scratch test (*p* = 0.625). Given our results and the previously mentioned research, it might be necessary to add targeted exercises for the external and internal rotators of the shoulder joint to TKD, BOX, and EBT interventions to enhance range of motion and promote beneficial adaptations in older women, such as longer fascicles and altered pennation angle [49].

For the 2 min step test, no significant improvements were found for the TKD, BOX, and EBT groups. Unlike the findings reported in [26], which reported significant improvements (*p* = 0.0004) in favor of a TKD intervention compared to a multicomponent training group in older women after 8 weeks of training with a total of 24 sessions and a duration of 60 min per session. Similar to that was reported in [41] in older women with arterial hypertension, where significant improvements were presented for a 2 min step test (*p* = 0.003) after a TKD intervention compared to an inactive control group with 12 weeks of training and a frequency of 3 times per week and a duration per session of 90 min. This is in line with that reported in [50] in older women who showed significant improvements in the 2 min step test (*p* < 0.05) after a 12-week EBT intervention with a frequency of 2 times per week. This contrasts with the findings reported in a recent study [26], which showed no improvements in the 2 min step test (*p* = 0.737) among older women after 16 weeks of TKD training. Despite the aerobic nature of our intervention, no significant improvements were found in the 2 min step test, a widely used field measure of cardiorespiratory fitness in older people [51]. One possible explanation is the relatively low number of training sessions (16 sessions over 8 weeks), which may have been insufficient to elicit detectable improvements, as supported by the higher-volume protocols in previous studies [26,41,52]. Moreover, the 2 min step test, while practical and safe, may lack the sensitivity of more robust laboratory assessments such as VO_2_ max or submaximal treadmill protocols. It is also important to note that our participants’ baseline values were already above normative standards for their age group [53], potentially creating a ceiling effect that limited measurable post-intervention improvements. Future studies should consider longer interventions and more sensitive assessments to better capture changes in cardiorespiratory fitness in this population.

### 4.1. Limitations and Strengths

The following were the study’s limitations: (i) inability to regulate food consumption (to ascertain participants’ eating patterns) and to ascertain participants’ dietary profiles, including their intake of protein, carbohydrates, fats, and micronutrients; (ii) difficulty extrapolating these findings due to the exclusion of older men; (iii) failure to examine physiological and/or biochemical factors; and (iv) selection bias, the participants who enrolled may have been more motivated or physically capable than the general older people, which could limit the generalizability of our findings. The following were some of the study’s strengths: (i) Comparing three physically active groups (TKD, BOX, and EBT) and randomly assigning participants at the outset improved the study’s internal consistency; (ii) using validated tests that are frequently used in training interventions for older people improved the study’s external validity; and (iii) using training programs tailored to older women’s characteristics decreased the risk of injury and improved adherence to the interventions.

### 4.2. Practical Applications

BOX and TKD adapted to the characteristics of older people can reduce the fall risk by improving stability and balance, which is in line with recent studies [18,26]; to do this, it is necessary to respect the basic principles of training and offer a load progression that respects the individual characteristics of older people to achieve the greatest benefits [15]. For its part, EBT provides significant improvements in MIHS (dominant and non-dominant hands), a relevant fact because higher MIHS is related to a lower risk of mortality from all causes in older people [54]. In addition, EBT is a low-cost, safe, accessible, and easy-to-implement intervention [16]. Finally, trainers and health professionals could systematize exercises and activities from Olympic combat sports (i.e., BOX and TKD) and EBT to offer physical activity programs aimed at older people that, in addition to achieving physical function, can generate greater adherence and satisfaction with the practice [16,17,18,19].

## 5. Conclusions

While there are no significant differences between groups or improvements in the other variables, the TKD and BOX interventions significantly decrease run time on the TUG test, while EBT increases muscle strength on the MIHS test for dominant and non-dominant hands in Chilean older women. Our findings support the positive effects of TKD, BOX, and EBT on the health status of older women and training regimens that may be most advantageous when used together. Future research could address the effects of these Olympic combat sports interventions combined with EBT.

## Figures and Tables

**Table 1:** Intervention dosage.

Program	Duration (Weeks)	Frequency (Weekly)	Time × Session (min)	Physical Exercise	Sets and Repetitions	Recovery	Intensity
TKD	1–2	2	60	Upper limb	3–8	2 min	OMNI-RES (5–8 points)
	3–5			Lower limb	4–8	2 min	
	6–8				4–12	90 s	
BOX	1–2		60	Upper limb	3–8	2 min	OMNI-RES (5–8 points)
	3–5			Lower limb	4–8	2 min	
	6–8				4–12	90 s	
EBT	1–4		60	Whole body			OMNI-RES (5–8 points)
	5–8				2–10 to 15	60 s	

TKD: taekwondo; BOX: boxing; EBT: elastic band training; min: minutes; s: seconds.

**Table 2:** Baseline anthropometric parameters and sociodemographic assessments of Chilean older women.

Variable	Assessment	TKD Group (*n* = 10)	BOX Group (*n* = 10)	EBT (*n* = 10)
Age (years)		72 (7.09)	71.8 (2.92)	71.6 (2.88)
Anthropometric parameters	Bipedal height (cm)	1.58 (0.05)	1.61 (0.07)	1.60 (0.05)
	Body mass (kg)	71.7 (12.0)	70.7 (13.0)	71.4 (19.4)
	BMI (kg/m^2^)	28.7 (8.1)	27.3 (7.3)	27.9 (9.2)
Academic level	Primary (%)	17	16	12
	Secondary (%)	11	13	16
	Bachelor (%)	3	5	7
	Postgraduate (%)	0	0	0
Civil status	Married (%)	32	16	18
	Separated (%)	7	9	4
	Widowed (%)	3	5	6
	Single (%)	0	0	0

BMI: body mass index; BOX: boxing; EBT: elastic band training; TKD: taekwondo. Age and anthropometric variables are presented as mean ± standard deviation. Sociodemographic variables are expressed as percentages.

**Table 3:** Time × group interaction on body composition and physical function before and after interventions in TKD, BOX, and EBT groups.

	Group	Before			After			Time × Group *p* Value	Time × Group F Value	ηp^2^
		Mean	SD	95% CI	Mean	SD	95% CI			
**Fat-free mass (kg)**	TKD	25.0	9.2	22.5–27.5	25.2	7.8	22.5–27.8	0.306	0.738	0.019
	BOX	24.4	7.0	22.0–26.7	24.7	7.3	22.6–26.9			
	EBT	22.4	4.9	20.4–24.3	21.0	3.9	17.9–24.1			
**Body fat percentage (%)**	TKD	39.5	9.2	33.7–45.4	39.4	7.8	34.4–44.4	0.091	2.588	0.143
	BOX	35.4	7.0	31.0–39.9	34.6	7.3	30.0–39.3			
	EBT	41.7	6.5	38.2–45.2	40.8	6.0	38.0–43.6			
**MIHS dominant hand (kg)**	TKD	18.4	4.3	11.4–25.5	19.0	3.1	11.7–26.2	<0.001	55.940	0.783
	BOX	16.2	4.6	13.0–19.3	15.4	3.6	12.3–18.4			
	EBT	19.1	4.9	15.6–22.6	23.7	6.1	20.4–27.0			
**MIHS non-dominant hand (kg)**	TKD	14.4	11.1	9.1–19.7	14.9	11.4	9.7–20.2	<0.001	29.180	0.652
	BOX	14.8	5.0	11.1–18.6	15.6	4.8	11.5–19.6			
	EBT	16.5	4.9	12.9–20.1	19.8	4.6	16.0–23.6			
**30 s chair stand (rep)**	TKD	12.5	8.3	11.2–13.8	15.0	8.3	13.8–16.2	0.211	1.636	0.095
	BOX	14.8	6.0	13.2–16.4	16.7	6.3	14.9–18.4			
	EBT	12.2	5.0	10.7–13.7	14.0	5.3	12.4–15.6			
**Back scratch (cm)**	TKD	−8.8	2.1	−11.2–6.5	−9.7	1.9	−13.0–6.3	0.084	2.665	0.139
	BOX	−11.3	2.5	−15.8–6.8	−14.3	2.7	−18.4–10.3			
	EBT	−9.2	2.1	−12.3–6.1	−8.0	2.2	−11.4–4.6			
**Sit-and-reach (cm)**	TKD	−14.8	3.7	−17.3–12.4	−13.2	5.3	−15.2–11.2	0.168	1.882	0.102
	BOX	−16.7	7.1	−21.0–12.4	−14.2	6.4	−18.9–9.5			
	EBT	−6.0	4.9	−7.8–4.2	−5.9	5.4	−7.7–4.1			
**TUG (seg)**	TKD	10.9	3.9	10.2–11.6	8.0	3.1	6.9–9.2	<0.001	21.770	0.584
	BOX	10.0	6.8	9.1–10.9	7.3	7.4	6.5–8.2			
	EBT	7.2	2.9	6.7–7.7	7.6	2.8	7.2–8.0			
**2 min step test (rep)**	TKD	95.8	1.1	79.8–111.9	101.0	1.8	82.8–119.2	0.166	1.982	0.113
	BOX	105.0	1.3	81.6–128.4	115.2	1.3	93.8–136.5			
	EBT	106.6	0.7	90.1–123.1	105.4	0.6	92.3–118.5			

BOX: boxing; EBT: elastic band training; MIHS: maximal isometric handgrip strength; TKD: taekwondo; TUG: timed Up-and-Go; SD: standard deviation; ηp^2^: partial eta square; 95% CI: 95% confidence interval.

## Data Availability

The datasets generated and/or analyzed during the current study are available upon reasonable request from the corresponding authors.
